# Supplementary material for: Identification and functional analysis of non-coding regulatory small RNA FenSr3 in Bacillus amyloliquefaciens LPB-18
Source: PeerJ. 2023 May 15;11:e15236. doi: 10.7717/peerj.15236 (PMC10194069; doi:10.7717/peerj.15236)
Supplement: Supplemental Information 4 [file peerj-11-15236-s004.zip › KO/CK-vs-T1_map/map00780.html]

KEGG PATHWAY: Biotin metabolism - Reference pathway


|  |  |
| --- | --- |
| **Biotin metabolism - Reference pathway** |  |

[
Pathway menu
| Organism menu
| Pathway entry
| Show description
| User data mapping
]

Biotin (vitamin H or vitamin B7) is the essential cofactor of biotin-dependent carboxylases, such as pyruvate carboxylase and acetyl-CoA carboxylase. Mammals cannot synthesize biotin, while in bacteria, fungi, and plants it is synthesized from pimelate thioester through different pathways. In E. coli and many organisms, pimelate thioester is derived from malonyl-ACP. The pathway starts with the methylation to malonyl-ACP methyl ester, followed by the fatty acid chain elongation cycle to form pimeloyl-ACP methyl ester, which is then demethylated to form pimeloyl-ACP [MD:M00572]. Pimeloyl-ACP is converted to biotin through the final four steps in the biotin bicyclic ring assembly, which are conserved among biotin-producing organisms [MD:M00123]. In B. subtilis, biotin is derived from pimeloyl-ACP formed by oxidative cleavage of long-chain acyl-ACPs [MD:M00573]. Some bacteria synthesize biotin from pimeloyl-CoA derived from pimelate [MD:M00577]. Biotin is covalently attached to biotin-dependent carboxylase by biotin protein ligase, also known as holocarboxylase synthase in mammals, to form an active holocarboxylase. After degradation of the biotinylated carboxylase into biocytin, it is further degraded by biotinidase to release free biotin, which is recycled in holocarboxylase synthesis. Biotin is catabolized by beta-oxidation of the valeric acid side chain or oxidation of sulfur in the heterocyclic ring.

|  |  |  |
| --- | --- | --- |
| Reference pathway | 184% 150% 122% 100% 82% 67% 55% | 图片下载 |
